# Value and engagement: what can clinical trials learn from techniques used in not-for-profit marketing?

**DOI:** 10.1186/s13063-022-06417-3

**Published:** 2022-06-02

**Authors:** E. J. Mitchell, K. Sprange, S. Treweek, E. Nixon

**Affiliations:** 1 Nottingham Clinical Trials Unit, Applied Health Research Building, University Park, University of Nottingham, Nottingham, NG7 2RD UK; 2 Health Services Research Unit, Health Sciences Building, University of Aberdeen, Aberdeen, AB25 2ZD UK; 3 Nottingham University Business School, Jubilee Campus, University of Nottingham, Nottingham, NG8 1BB UK

**Keywords:** Randomised trials, Marketing, Value, Recruitment, Retention

## Abstract

Marketing is a core business function in commercial companies but is also frequently used by not-for-profit organisations. Marketing focuses on understanding what people value to make choices about engaging with a product or service: a concept also key to understanding why people may choose to engage with a clinical trial. Understanding the needs and values of stakeholders, whether they are participants, staff at recruiting sites or policy-makers, is critical for a clinical trial to be a success. As many trials fail to recruit and retain participants, perhaps it is time for us to consider approaches from other disciplines. Though clinical trial teams may consider evidence- and non-evidence-based recruitment and retention strategies, this is rarely done in a systematic, streamlined way and is often in response to challenges once the trial has started. In this short commentary, we argue the need for a formal marketing approach to be applied to clinical trials, from the outset, as a potential prevention to recruitment and retention problems.

## Marketing in the not-for-profit sector

In 2019, the Royal Observatory in Greenwich wanted to extend the reputation of its telescopes and reach new audiences to improve public understanding of astronomy. As a non-profit organisation, the budget was just £550 [1]. Via social media, press coverage and partnerships with other organisations, its coverage of the lunar eclipse in 2019 had more video views than any other event from a museum globally. The campaign reached more than 310,000 people as well as driving 360,000 visits to the observatory’s website and an 18% growth in sign-ups to its newsletter. Behind the campaign was a simple but vital factor; the Royal Observatory looked at their organisation from the point of view of their audience and, crucially, understood what it was their audience really valued. The initiative was a success because the Royal Observatory understood their audience: they knew what was important to that audience and made use of existing collaborations to support engagement. Having a small budget is rarely an advantage, but neither is it the insurmountable barrier we often think it is.

Marketing in healthcare contexts has a dubious reputation, often associated with profit-making and manipulative techniques that jar with the principles of welfare underpinning the UK’s National Health Service (NHS). Yet, marketing is also used by not-for-profit organisations to improve services, attract resource such as funds or volunteers and increase engagement in their activities [2, 3]. Simply put, marketing seeks to understand consumers’ needs, desires and values, enabling people to make informed choices. By looking at your organisation from the point of view of your audience—be they donors, participants, investors or the public—marketing activities help achieve the organisational mission in cost-effective ways that minimise wastage. Marketing emphasises *what people value* when choosing goods and services and the quality of their experiences [4]. Indeed, many of us choose to repeatedly purchase a particular brand or use a particular service because it continues to provide us with an experience or benefit that we value.

## Understanding what people value in clinical trials

What happens if we consider a clinical trial as a service, or more precisely, as an experience people value? While the design and development of a clinical trial largely focuses on the science, the recruitment and retention of trial sites and participants is akin to a business or organisation attracting people to a particular service or trying to reach a new audience. Those businesses and organisations will have thought about *who* they need to engage with, *why* they want to do this, *how* best to reach them, *what* these people value and what might make them stay. They will, in short, have done their homework. If this sounds familiar, that is because it is: it sounds a lot like trial recruitment and retention. For example, understanding *who* needs to be involved in a clinical trial, whatever their role or involvement, is crucial. This concept underpins the National Institute for Health Research (NIHR) INCLUDE initiative, which aims to help trial teams think more carefully about *who* should be in their trial to make sure trials better serve groups who have been under-served by research in the past [5]. The INCLUDE Ethnicity Framework presents questions trial teams should ask themselves when considering the ethnic groups who must be included in the trial for the results to be applicable [6]. Knowing your audience, the people you wish to engage, is the first step in *planning* recruitment and retention, which is an essential activity. Getting this right is critical if we are to answer important research questions that could impact the health and wellbeing of patients in the future. It is suggested that less than half of all trials meet their target, with or without a funded or non-funded extension [7, 8].

## Adopting a marketing approach to clinical trials?

Over a decade ago, Francis et al. [9] argued that clinical trials can benefit from adopting principles and techniques used in the discipline of marketing, in other words engaging in some research and planning before attempting to recruit and retain trial participants. For example, trialists need to develop strategies to make the trial attractive to potential recruiting sites and participants and initiate ways in which they remain engaged and involved in the trial until its completion [10–14]. The challenge for trialists is *how* to do this well. Although often considered during the funding application or set-up phase of a trial, even highly experienced teams are reactive and focus on implementing additional strategies when recruitment and retention problems begin, as a *treatment*, rather than fully considering much earlier on, how to *prevent* problems.

How do not-for-profit organisations use marketing to recruit and retain participants well? We know from marketing scholarship that face-to-face interpersonal contact is by far the most effective recruitment tactic and that this is further increased when the person recruiting is known by the person being recruited [3] . Referrals, or consumer-to-consumer advocacy, are also highly influential. When members of an online support group for life-threatening diseases posted about a clinical trial on a new drug to treat leukaemia, the trial managers were overwhelmed with demand from patients seeking to participate [4]. When a community development programme were struggling to recruit participants to free courses, they learned that the residents they needed to reach were ignoring their posters and flyers because they were suspicious of anything described as ‘free’ by people who appeared to be strangers, assuming there had to be a catch [15].

Viewing clinical trials from a marketing perspective also emphasises the importance of the *experience* of the trial for everyone involved, from participants to recruiting sites and wider stakeholders. This requires careful consideration of the unique *value* a specific trial holds for each stakeholder group and not just the trialists or eventual beneficiaries. Trialists should, from the outset, consider *who* their stakeholders are, e.g. potential participants, actual participants, staff at recruiting sites and the wider healthcare and academic communities, and learn *what matters to them*, to enhance the value of the trial experience for them. Active patient and public involvement and engagement (PPIE) and consideration of equality, diversity and inclusion is of particular importance too, since what matters to some groups of people may be very different to others. Understanding stakeholders’ *different* motivations helps improve retention by effective use of marketing techniques that minimise disenchantment—where participants become dissatisfied because the experience falls short of what they imagined it to be. It is marketing when trialists try to ensure stakeholders’ different reasons for involvement are satisfied, when accurate role descriptions and site tours are provided so new recruits have realistic expectations, and when tailored messages or other interventions are designed to help stakeholders feel appreciated throughout a trial. A ‘one size fits all’ approach is unhelpful when considering who you want to attract and what might attract them to participating and remaining in a clinical trial.

A more systematic and sensitive understanding of people’s values and motivations to engage is a crucial first step for developing and implementing effective and creative recruitment and retention strategies. Contemporary marketing strategies also apply models from behavioural science such as ‘nudging’ [16] to encourage positive changes in behaviour, with particular success in public health contexts. When the Brazilian government health department were struggling to encourage women of the Amazonian rainforests to have regular cervical smear tests, a particular orchid that flowers once a year was distributed to 5000 women as a ‘nudge’ to act as a calendar and remind them to attend a clinic [17]. Understanding the cultural resonance of nature for sustenance and medicine among these local communities was key to developing an effective intervention.

While trialists initiate recruitment and retention strategies at the beginning of a trial, this may often be done on an ad-hoc basis and can vary depending upon a variety of factors including the experience of the trialist and the time available. Drawing upon marketing literature, Francis et al. [9] developed a reference model that incorporates four key areas considered important for a clinical trial: (i) maintaining engagement, (ii) building brand values, (iii) making the sale and product and (iv) market planning. Though the commercial-style language may impede implementation of this model, we think that some of these activities may well already be informally employed by trialists. However, there is a lack of evidence about whether and how trialists *formally* implement a marketing approach to their clinical trial and, importantly, its impact on recruitment and retention. McDonald et al. [18] retrospectively applied this model to three trials which showed potential benefits and feasibility of the approach. However, to our knowledge, there has been no prospective evaluation of applying the marketing approach to non-commercial clinical trials.

Recruiting and retaining participants in a clinical trial are two of the most important process endpoints in a trial. Indeed, in a study designed to develop performance metrics for clinical trial sites, recruitment and retention were flagged as key trial process indicators, along with data quality [19]. Evaluating strategies to improve the processes of clinical trial recruitment and retention is vital in order to improve trial efficiency and reduce research waste [20, 21].

There are some examples of where trials have understood the values and needs of their stakeholders, leading to successful recruitment and retention. In the Football Fans in Training (FFIT) trial, overweight men were recruited via a wide advertising strategy to participate in a weight-loss programme [22]. The weight-loss programme was conducted in Scottish professional football clubs and participants had behind-the-scenes access to the clubs, which participants valued. The CRASH-2 trial recruited ~ 20,000 adult trauma patients in forty countries to investigate the effect of tranexamic acid on death [23]. Here, it was particularly important to engage with a substantial number of clinicians, across the world, and understanding what would lead them to randomising a patient into the trial was crucial. In the CLOTHES trial, which investigated silk garments for children with eczema, retention of participants, particularly in the ‘usual care group’ (i.e. control), was facilitated by maintaining regular contact with families and offering the garments at the end of the trial, utilising a waiting-list control design [24]. The BEEP trial, comparing strategies for the prevention of eczema in young children, had an 87% retention rate 2 years after recruitment, with no face-to-face contact in between. A variety of strategies were used including providing high-street shopping vouchers, contact via text messages, sending newsletters to families to keep them updated about the trial and sending birthday cards [25]. Understanding how best to recruit and retain participants is also highly relevant for other study designs such as cohort or longitudinal studies [26].

If we are to answer important health questions to improve the health and wellbeing of the public, and want to do this efficiently, perhaps it is time to look outside the world of clinical trials to learn from other disciplines, such as not-for-profit marketing. Research into *how* to take lessons from marketing and apply them to clinical trials is needed, as is evaluation of the impact of this. Looking at recruitment and retention in new ways must be part of a strategy to reduce research waste and inefficiencies in trials. No trial, whatever its budget, can afford waste and inefficiency: patients and the public certainly cannot.

## Data Availability

Not applicable.

## Abbreviations

BEEP trial: Barrier enhancement for eczema prevention study
CLOTHES trial: Clothing for the relief of eczema symptoms trial
CRASH-2 trial: Clinical Randomisation of an Antifibrinolytic in Significant Haemorrhage trial
NHS: National Health Service
NIHR: National Institute for Health Research
PPIE: Patient and public involvement and engagement
